# Operando tracking of oxidation-state changes by coupling electrochemistry with time-resolved X-ray absorption spectroscopy demonstrated for water oxidation by a cobalt-based catalyst film

**DOI:** 10.1007/s00216-021-03515-0

**Published:** 2021-07-17

**Authors:** Chiara Pasquini, Si Liu, Petko Chernev, Diego Gonzalez-Flores, Mohammad Reza Mohammadi, Paul Kubella, Shan Jiang, Stefan Loos, Katharina Klingan, Vadim Sikolenko, Stefan Mebs, Michael Haumann, Paul Beyer, Luca D’Amario, Rodney D. L. Smith, Ivelina Zaharieva, Holger Dau

**Affiliations:** 1 Department of Physics, Freie Universität Berlin, Arnimallee 14, 14195 Berlin, Germany; 2 Department of Chemistry - Ångström Laboratory, Molecular Biomimetics, Uppsala University, Lägerhyddsvägen 1, 75120 Uppsala, Sweden; 3 Centro de Electroquímica y Energía Química (CELEQ) and Escuela de Química, Universidad de Costa Rica, San José, 11501 2060 Costa Rica; 4 Department of Physics, University of Sistan and Baluchestan, Zahedan, 98167-45845 Iran; 5 Fraunhofer Institute for Manufacturing Technology and Advanced Materials (IFAM), Winterbergstraße 28, 01277 Dresden, Germany; 6 Karlsruhe Institute of Technology, Karlsruhe (KIT), Adenauerring 20, 76131 Karlsruhe, Germany; 7 Department of Chemistry, University of Waterloo, 200 University Ave. W, Waterloo, ON N2L 3G1 Canada

**Keywords:** Electrocatalysts, Time-resolved X-ray absorption spectroscopy, Transition metal oxides, Water oxidation

## Abstract

Transition metal oxides are promising electrocatalysts for water oxidation, i.e., the oxygen evolution reaction (OER), which is critical in electrochemical production of non-fossil fuels. The involvement of oxidation state changes of the metal in OER electrocatalysis is increasingly recognized in the literature. Tracing these oxidation states under operation conditions could provide relevant information for performance optimization and development of durable catalysts, but further methodical developments are needed. Here, we propose a strategy to use single-energy X-ray absorption spectroscopy for monitoring metal oxidation-state changes during OER operation with millisecond time resolution. The procedure to obtain time-resolved oxidation state values, using two calibration curves, is explained in detail. We demonstrate the significance of this approach as well as possible sources of data misinterpretation. We conclude that the combination of X-ray absorption spectroscopy with electrochemical techniques allows us to investigate the kinetics of redox transitions and to distinguish the catalytic current from the redox current. Tracking of the oxidation state changes of Co ions in electrodeposited oxide films during cyclic voltammetry in neutral pH electrolyte serves as a proof of principle.

**Graphical abstract:**

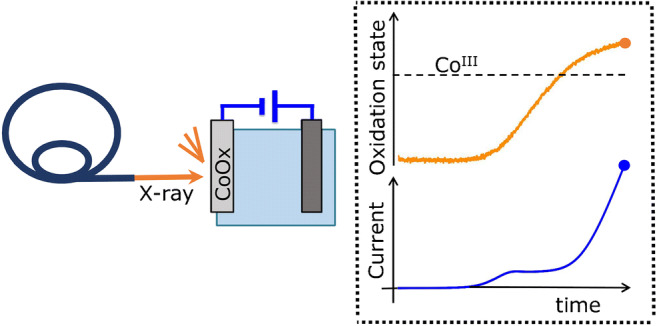

**Supplementary Information:**

The online version contains supplementary material available at 10.1007/s00216-021-03515-0.

## Introduction

The transition from fossil fuels towards renewable energy sources requires massive efforts in technological developments. In this context, synthetic (non-fossil) fuels are of high interest, with electrocatalytic water oxidation emerging as a critical key process [1]. First-row transition metal oxides are amongst the best choices as electrocatalysts for water oxidation due to their proven OER activity and potential in low-cost large-scale application [2, 3]. According to our current knowledge, during the catalytic splitting of H_2_O into protons, electrons, and molecular oxygen, catalysts accumulate oxidizing equivalents via changes in metal oxidation state [4–10]. The stored oxidizing equivalents are then used to oxidize water molecules. This is also observed in the biological water oxidation reaction. For example, the proposed reaction mechanism for OER on cobalt-based catalysts involves two proton-coupled electron transfer reactions, linked to Co^II^ → Co^III^ → Co^IV^ oxidation, before onset of the rate-limiting O–O bond formation [5, 7, 11, 12].

To develop a strategy to improve catalytic activity and stability, a better understanding of the mechanism of water oxidation is needed. This can be gained by systematic studies of metal oxidation state changes during electrocatalysis and a very well-suited method for this is hard X-ray absorption spectroscopy (XAS). XAS at the metal K-edges is a bulk-sensitive method that can be used to determine the metal oxidation state of a specific atom type with a precision of up to 0.1 oxidation-state units [13, 14]. In X-ray absorption spectroscopy, the energy region of about 50 eV, where a sharp increase of the absorption is detected, is known as X-ray absorption near-edge structure (XANES) [15], and from the energy position of this sharp increase, the average oxidation state of the metal can be derived.

The utility and information content of XAS combined with electrochemistry was demonstrated, inter alia, by the quasi-in situ, freeze-quench method. The freeze-quench method consists in freezing the catalyst during operation for subsequent analysis [8, 12]. This method is associated with all the advantages of typical XAS experiments carried out at cryogenic temperature (e.g., 20 K), where the spectral resolution is higher and the radiation damage during the XAS measurements can be better controlled, but captures a fixed state of the catalyst and has no time resolution.

To obtain a time resolution, operando experiments are required, where the XAS spectra are measured during electrochemical experimentation [16]. (We note that “operando XAS” is also referred to as “in situ XAS.”) For example, cyclic voltammetry (CV) is a powerful electrochemical technique capable of providing insights in both energetics (redox potentials) and kinetics (diffusion limitations, rate constants) of a chemical reaction [17]. The main factor limiting the combined use of operando XAS and electrochemical experiments with typical CV scan rates is the data collection time required for a complete XANES spectrum, which decreases the temporal resolution of the technique. Significant progress has been achieved in the technical development of this approach in recent years [18, 19]. Typically, the studies approaching operando XAS measurements aim at quick collection of an entire spectrum, either by using energy-dispersive XAS [20, 21] or quick scanning extended X-ray absorption fine-structure (QEXAFS) spectroscopy [22–24]. Also, pump and probe XAS combined with high brilliance beamlines or X-ray free-electron lasers (XFEL) allows the investigation of processes in the sub-second timescale [25–27].

All the above-mentioned techniques for operando XAS data collection require special technical equipment, which is available only at few beamlines in the world. Moreover, signal-to-noise problems hamper the investigation of many electrocatalysts of interest, mainly because a sensitive X-ray fluorescence detection scheme cannot be implemented easily. On bending magnet beamlines, equipped with a standard double-crystal monochromator, the acquisition time for fluorescence-detected X-ray absorption spectra (including the EXAFS region) may take from minutes up to hours. Specific capabilities depend on the technical characteristics of the synchrotron beamline and on the strength of the X-ray fluorescence of the catalyst material, which is determined by factors such as the thickness of the investigated metal oxide film.

Here, we present an alternative approach that provides a time resolution of 1 ms, for amorphous metal oxide catalyst films with a thickness of about 90 nm. The approach consists in recording the X-ray fluorescence signal at a single-energy [28] and then converting this signal into a number that describes the mean metal oxidation state quantitatively, with a method that enables tracking of fast temporal changes. An electrochemically deposited Co oxide catalyst (CoCat) operated at neutral pH [29] is used to demonstrate a proof of concept and highlight the several factors that must be taken into account for obtaining a reliable oxidation-state estimate. Using the CV experiment as an example, we analyze the information content and restrictions of the single-energy time-resolved operando XAS approach.

## Materials and methods

### Catalyst film deposition

CoCat films were prepared by anodic electrodeposition following an established protocol [29, 30] with minor modifications: a potential of 1.05 V_NHE_ was applied in a 0.1-M phosphate buffer (KPi) at pH 7 containing 0.5 mM Co(NO_3_)_2_ solution. Sample preparation, as well as electrochemical experiments, were performed in a single-compartment three-electrode cell, using a Pt grid (2 × 2 cm^2^, 90% purity) as a counter electrode and an Ag/AgCl (saturated) as a reference electrode. The working electrode was a 0.1-mm-thick glassy carbon sheet (Hochtemperatur-Werkstoffe GmbH, 2 cm^2^ active area). Before use, glassy carbon substrates were sonicated in ethanol and water for 10 min each. The electrolyte solution for all experiments was prepared using Milli-Q water ( > 18 MΩ cm).

### Operando X-ray absorption spectroscopy

X-ray absorption spectroscopy measurements were performed at the beamline KMC-3 [31] at BESSY II synchrotron (Helmholtz-Zentrum Berlin, HZB). The XAS experimental station at KMC-3 is operated in the framework of a cooperation contract by the Freie Universität Berlin (workgroup of H. Dau) and the HZB. Electrochemistry took place in a three-electrode self-made Teflon cell, where the working electrode was attached as a window to the wall (Supplementary Information (ESM), Fig. S1). The X-ray excitation beam hits the back of the working electrode. An out-of-focus geometry assured a beam size at sample of 11 × 2 mm, because the large area prevents radiation damage problems. The Co fluorescence was filtered by a Fe metal foil (10 μm thick, 99.99+% purity, Goodfellow) and then collected, at an angle of ca. 90° from the incoming beam direction, by a scintillation detector (51BMI/2E1-YAPNeg, Scionix) using a photomultiplier operated at 0.9 kV. The outgoing current signal was converted to a voltage with a 1-MΩ resistor, amplified, and then recorded by a potentiostat (BioLogic SP-300) which at the same time performed electrochemistry using the EC-Lab 11 software.

For energy calibration, the pre-edge feature of each edge scan was aligned: first, the derivative of the edge scan was calculated, then a Gaussian function was fitted through it and the peak of the Gaussian, corresponding to the center of the pre-edge rise, was shifted to 7708 eV.

## Results

### XANES versus single-energy X-ray fluorescence

**Fig. 1:**
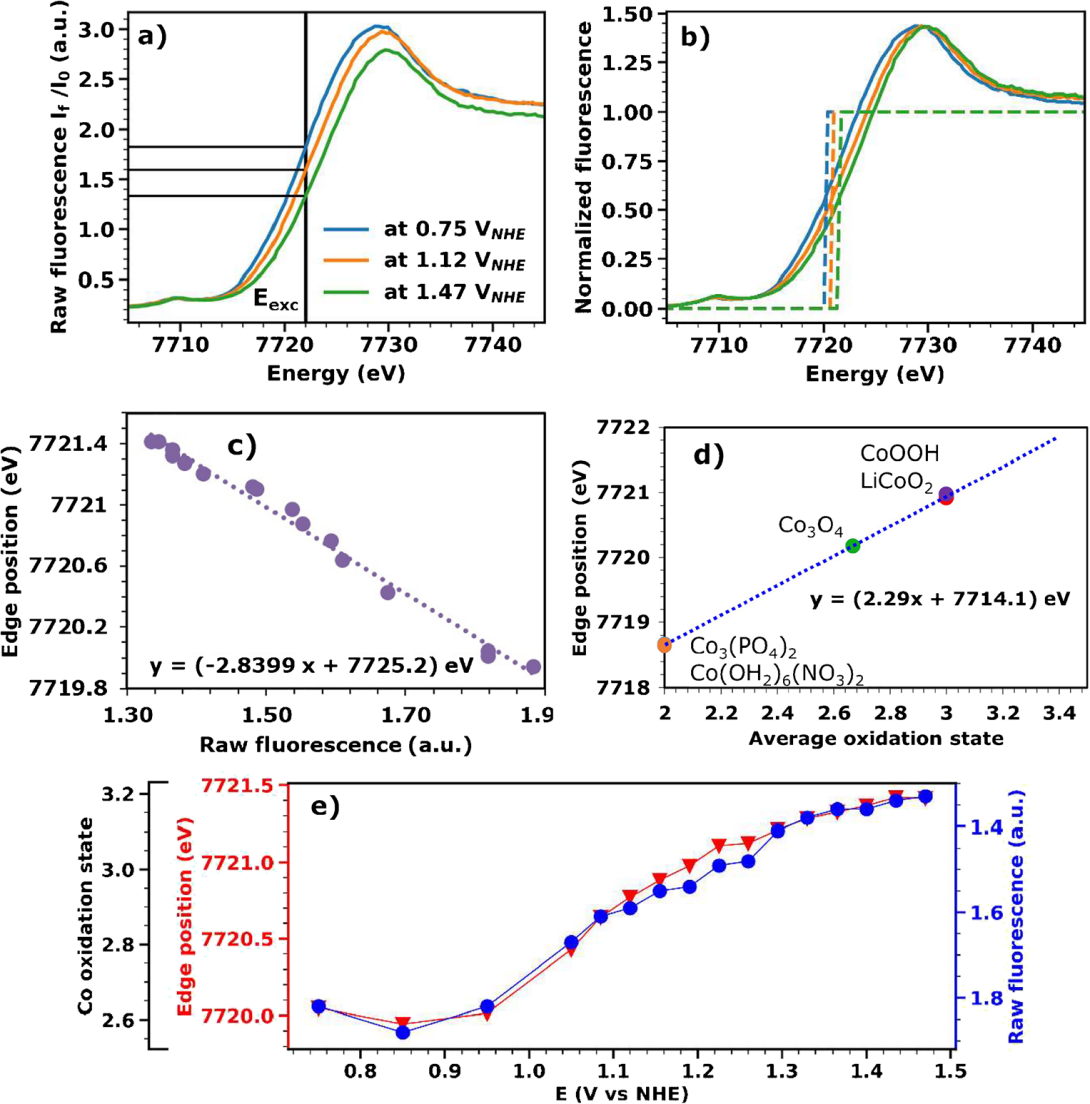
Conversion of the X-ray fluorescence signal intensity to Co oxidation states for a CoCat film operated in 0.1 M KPi solution at pH 7. **a** Three X-ray edge spectra (XANES) recorded at different electric potentials. The entire dataset consists of 16 spectra collected in the potential range between 0.75 and 1.47 V_NHE_ and is shown as ESM (Fig. S2). The fluorescence intensities for excitation at E_exc_ = 7722 eV in the raw, non-normalized spectra are indicated by black horizontal lines. **b** Three normalized XANES recorded at different electric potentials. The edge energies obtained by the integral method [14] are indicated by the dotted lines. **c** Calibration curve to convert the “raw” fluorescence intensity detected for excitation at 7722 eV to edge positions determined by the integral method. **d** Calibration line relating the edge positions determined by the integral method to the Co oxidation state, constructed using reference compounds with known structure and oxidation state (reference spectra shown in ESM Fig. S3). **e** Co oxidation states estimated from the edge positions determined from normalized XANES spectra (in red) or from the “raw” fluorescence signal detected for excitation at 7722 eV (in blue). The left y-axis scale (edge position) and the right y-axis scale (raw fluorescence) are chosen such that at the lowest and highest potential the data point for edge positions and raw fluorescence coincide

XAS spectra were collected in the X-ray energy range from 7580 to 8260 eV and used to estimate the Co oxidation state for CoCat films equilibrated at 16 different potentials between 0.75 and 1.47 V_NHE_. Each spectrum was recorded within 5 min after 1 min equilibration. During the equilibration time, a stable current is reached, which ensures that the oxidation state changes induced by the potential increase have been largely completed. The recorded fluorescence signal divided by the incoming beam intensity (called here “raw fluorescence,” Fig. 1a) was further processed following a standard procedure of energy calibration, background subtraction, and normalization, to obtain the normalized spectra used to estimate the Co oxidation state (Fig. 1b).

The experimental setup was designed to accommodate a standard volume of electrolyte (50 mL), in order to preserve appropriate electrochemical conditions. Electrolyte layers thicker than a millimeter cannot be penetrated by X-rays, necessitating measurement of the Co K_α_ line in X-ray fluorescence mode. The incoming X-ray beam passes through the 100-μm-thick conductive glassy carbon substrate to reach the catalyst film; after which, the Co fluorescence is emitted and collected at an angle of ca. 90° from the incoming beam direction. The scheme of the experimental setup is shown in the ESM Fig. S1. A linear relationship between the absorption and fluorescence signals can be assumed, because, in our experiments, the total absorption by the Co ions in the thin catalyst film is low [32].

For first-row transition metals, the position of the X-ray K-edge shifts to higher energies for increasingly positive formal oxidation state of the metal ion, typically by 2–3 eV per oxidation-state unit [14, 33]. For each first-row transition metal in a metal-ligand environment that contains hard ligands only (O, N), an approximately linear relation between X-ray edge position and formal metal oxidation states is found [12, 14, 34], as exemplarily shown for Co compounds in Fig. 1d. To increase the accuracy of the experiment, the X-ray edge position was determined using a suitable integration procedure of the entire edge rise, named the integral method (details in [14] and a comparison to other methods in ESM Note 1).

For the CoCat film investigated here, the edge energies increase continuously from about 7720.0 eV at 0.85 V_NHE_ to 7721.4 eV at 1.47 V_NHE_ (Fig. 1e, data points and y-axis in red). The slight decrease between 0.75 and 0.85 V_NHE_ is likely due to an insufficient equilibration time at the first investigated potential. Using the calibration line in Fig. 1d, the edge energies translate to a mean Co oxidation state (black y-axis in Fig. 1e) that ranges from about + 2.6 at the lowest potentials to about + 3.2 at the highest potentials. We emphasize that the non-integer oxidation state most likely describes a valence mixture, e.g., + 2.6 indicating 40% of all Co ions with an electronic structure corresponding to Co^2+^ and 60% with a Co^3+^ electronic structure.

### Procedure for the conversion of the X-ray signal into an oxidation state value

The approach for the estimation of metal oxidation states from XANES spectrum usually requires the collection of XAS spectra over an energy range of several hundred eV; the energy range is determined by the need for precise normalization of the edge jump to unity. Typically, this requires data collection times of several minutes, at a standard bending magnet beamline, or of several seconds, at a high-flux beamline with rapid scan capabilities. Thus, it renders time-resolved detection of oxidation-state changes problematic. In the following, we show that the fluorescence signal recorded at a single excitation energy in the middle of the absorption edge can be used to estimate the metal oxidation state. This enables dramatically improved time resolutions in the order of millisecond.

The procedure involves a series of steps and the construction of two calibration lines:

1. Acquire two XANES spectra with the same applied potential, one before and one after each time-resolved experiment. If they are not identical, secondary processes (e.g., film dissolution) may have affected the experiment; for details on possible solutions, see the next section.
2. Perform the electrochemical experiment while illuminating the sample with an X-ray beam at fixed energy. The energy of the X-ray beam is chosen to ensure highest amplitude of fluorescence changes (around the middle of the absorption edge, here E_exc_ = 7722 eV).
3. After the electrochemical experiment, collect at least two (three suggested) XANES spectra at the extremes of the potential range of interest, to serve as reference spectra.
4. In the reference measurements, divide the fluorescence signal by the incoming beam intensity and note the fluorescence value corresponding to the selected energy (E_exc_); here, this value is called “raw fluorescence signal” and will constitute the x-axis of the first calibration line.
5. Process the reference spectra by performing energy calibration for each of them (details in Supplementary Note 2), subtracting a linear background (usually obtained via linear fit in the low-energy region preceding the absorption edge) and normalizing to unity the fluorescence signal after the edge (via division of the spectrum by a polynomial fit or a spline calculated in the energy region above the absorption edge).
6. Calculate the edge energy by using the integral method [14] and build a calibration line relating the edge energy to the raw fluorescence signal (Fig. 1c). A new calibration line should be constructed for each change of experimental conditions, such as X-ray fluorescence detector position, detection settings, or sample thickness.
7. Collect XANES spectra for a series of reference compounds with known oxidation state, which contain the same metal and the same type of ligand as the material of interest (in our case Co coordinated to oxygen). Process the spectra as explained above, perform energy calibration, and calculate their edge position.
8. Use the reference compounds data to construct a second calibration line linking the edge position to the metal oxidation state (Fig. 1d, XANES spectra as ESM Fig. S3). For most metals, a linear dependence of the edge position on the metal oxidation state is expected [35].
9. Use the two calibration lines to convert the raw fluorescence signal recorded during the electrochemical experiment first to an edge position and then to an average oxidation state.

As a proof of principle, we use the series of XANES spectra collected at different potentials (Fig. 1a), to illustrate that virtually the same information could be obtained if only two XANES spectra were collected at the lowest and highest potentials, while at the other potentials only one fluorescence value was recorded, corresponding to the fluorescence emitted after excitation with X-rays with energy 7722 eV. The fluorescence value at E_exc_ = 7722 eV, after division by the incoming beam intensity, is shown as a blue line in Fig. 1e. The highest and lowest potentials act as reference measurements; therefore, the axes are chosen in a way that the first and last data point for the blue and red curves coincide. The very small differences between red and blue curves suggest that, even if only two XANES spectra are collected and processed (blue curve scenario), the Co oxidation state at intermediate potentials can be estimated with similar accuracy as if a XANES spectrum was collected at each potential (red curve scenario).

**Fig. 2:**
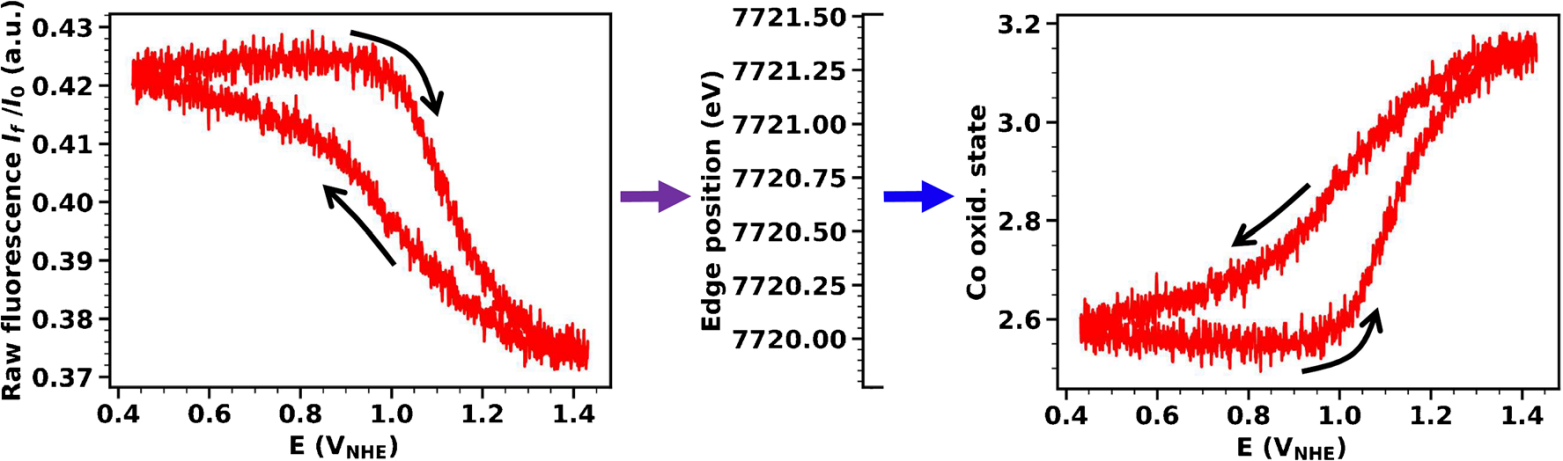
Raw fluorescence signal obtained by illuminating the catalyst with X-ray energy fixed at E_exc_ = 7722 eV while performing a cyclic voltammetry experiment (scan rate 10 mV s^−1^). The X-ray fluorescence signal was divided by the incoming beam intensity. Calibration curves from Fig. 1 (panels c and d) are used to convert the raw fluorescence to an edge position and then to a Co oxidation state. Data from a single CV scan are smoothed with a moving average across 15 data points (1 data point corresponds to 10 ms)

The described procedure can facilitate fast detection of metal oxidation-state changes for various types of electrochemical experiments, such as (i) potential jumps (sudden, stepwise change of the working electrode potential) which can provide information about the reaction kinetics (rate constants) of metal redox-state changes and possibly allow for detection of transiently formed reaction intermediates relating, e.g., to deprotonation or structural changes [27, 36] and (ii) redox changes during cyclic voltammetry [17]. In the following, we will focus on the use of the method to follow the Co oxidation state during a CV (Fig. 2), and we will illustrate possible pitfalls and strategies to deal with them when recording time-resolved operando X-ray absorption data.

### Irreversible structural changes and sample dissolution

The described procedure is sensitive to several error sources. Some of them, like the energy calibration of the recorded XANES spectra and possible radiation damage of the sample are inherited from the XAS method itself and are briefly discussed in the ESM (Notes 2 and 3, Figs. S4–S6). Possible sources of additional error are irreversible structural changes during the measurements as well as sample dissolution. These will be discussed in more detail in the following.

**Fig. 3:**
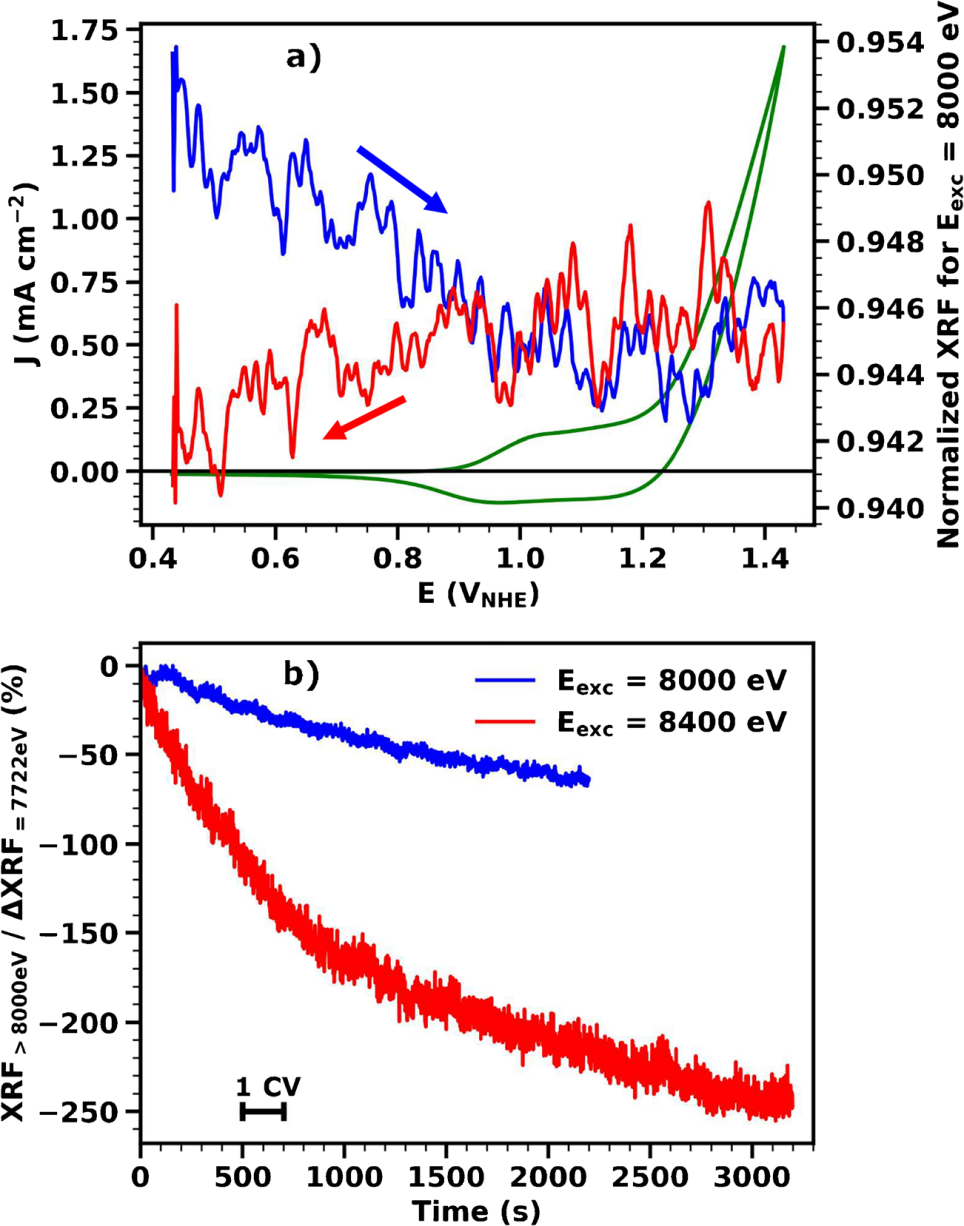
Time-resolved study of film dissolution for a CoCat sample (thickness 90 nm, corresponding to 12.5 mC cm^−2^) operated in 0.1 M KPi at pH 7. The X-ray fluorescence is excited at energies above the Co absorption edge. At these excitation energies, the fluorescence signal is proportional to the quantity of material in the sample, i.e., is only sensitive to dissolution phenomena. **a** Current density (green line, left axis) during the CV. Raw fluorescence signal (XRF) excited at 8000 eV is mapped on the right axis and shown with blue color for the anodic scan and with red for the cathodic scan. The arrows indicate the scan direction. The fluorescence was first divided by the incoming beam intensity and then normalized by subtracting the background level and dividing it by the total edge jump. In this scale, 1 represents the value of fluorescence at the beginning of the experiment, before dissolution, and 0 the value of fluorescence for a completely dissolved sample. The data represent the average of 9 CVs. **b** Fluorescence changes caused by film dissolution, measured at two different exemplary excitation energies recorded during a series of CVs as a function of time. Fluorescence intensity excited at E_exc_ > 8000 eV is shown as a percentage of the change in intensity due to oxidation state changes, which was calculated from the fluorescence recorded during a CV with an excitation energy of 7722 eV. The black horizontal bar indicates the timescale of one CV.

To check for the possible presence of undesired processes that can affect the oxidation-state assignment, XANES spectra at the same electrochemical conditions (e.g., same potential applied) should be collected both before and after the time-resolved operando experiment. If the two spectra are not identical, different cases should be considered:

1. Change in the edge shape. An example of such behavior is shown in the ESM Fig. S7. This indicates that the sample is being structurally modified during the experiment. A preliminary electrochemical treatment of the sample is needed to ensure that the structural changes are completed before the beginning of the operando experiment, in order to obtain reliable results.
2. Change in the edge position. An example of such behavior is shown in Table 1. At parity of electrochemical conditions, the metal oxidation state and, thus, the edge position observed for a sample is expected to be repeatable. A difference in the position of absorption edges measured before and after the experiment suggests that the oxidation state changes, observed during the operando experiment, are not fully reversible. This does not affect the reliability of the calculated oxidation state, but it should be considered when interpreting the experimental data.
3. Decrease in the fluorescence level at X-ray energy well above the absorption edge. An example of such behavior is shown in Fig. 3. This indicates that a part of the fluorescence-emitting sample has been lost during the experiment. Two main reasons can cause this effect: (i) the sample is detaching from the substrate, in which case the experiment cannot lead to reliable conclusions and different substrates should be tested and (ii) the sample is gradually dissolving in solution, during the electrochemical experiment. In the latter case, it is possible to correct the recorded fluorescence signal and compensate for the dissolution using the procedure outlined in the following.

**Table. 1:** Estimation of sample dissolution and irreversible oxidation state changes during CVs by XANES analysis. The corresponding absorption edges are shown in Fig. 4a. The percentage of dissolution was obtained via a fit of the 7822 to 8000 eV region with a horizontal line, after division by the incoming beam intensity and background subtraction. The edge position was obtained via the integral method, after edge normalization and alignment. The oxidation state was obtained using the calibration curve shown in Fig. 1d

	Dissolved film (%)	Edge position (eV)	Average Co ox. state
At 0.5 V before CVs	0	7720.09	2.62
At 0.5 V after 1 CV	3.0	7720.15	2.64
At 0.5 V after 2 CVs	5.3	7720.22	2.67
At 0.5 V after 3 CVs	7.6	7720.24	2.68
At 0.5 V after 13 CVs	12.5	7720.33	2.72
At 1.4 V after 13 CVs	13.8	7721.78	3.36

It is known that CoCat is not stable at pH 7, but slowly dissolves in Co-free electrolyte [37, 38]. Thus, the amount of Co atoms that contribute to the X-ray fluorescence is reduced and the recorded signal decreases. During an ordinary operando experiment, the fluorescence decrease due to dissolution is superimposed on the decrease due to metal oxidation. To distinguish the two phenomena, additional experiments are needed. One approach is to excite X-ray fluorescence at energies well above the absorption edge, where the EXAFS modulations are negligible and the fluorescence intensity does not depend on the structure or oxidation state, but solely on the amount of Co in the sample.

In this study, we performed two experiments, in which the X-ray fluorescence was recorded operando during a sequence of CVs, while the energy of the X-ray beam was set well above the Co absorption edge (E_exc_ > 8000 eV, Fig. 3). A clear fluorescence decrease is seen at potentials below 1.1 V, suggesting that the rate of dissolution is potential dependent, and above this potential, the signal is stable. In Fig. 3b, the impact of dissolution is estimated. The decrease due to dissolution (measured at E_exc_ > 8000 eV) is presented as a percentage of the total decrease due to oxidation state changes (measured at E_exc_ = 7722 eV) during a CV. It can be observed that the effect of sample dissolution is comparable in magnitude to the effect of oxidation state changes, but becomes less severe with increasing operation time. Large variations in the dynamic of film dissolution between different samples are possible and the difference between the two examples, shown in Fig. 3b, is most likely attributable to samples variability. Measurements at an excitation energy above the edge might be needed, at the beginning of every experiment, until a relatively stable state of the catalyst is reached.

**Fig. 4:**
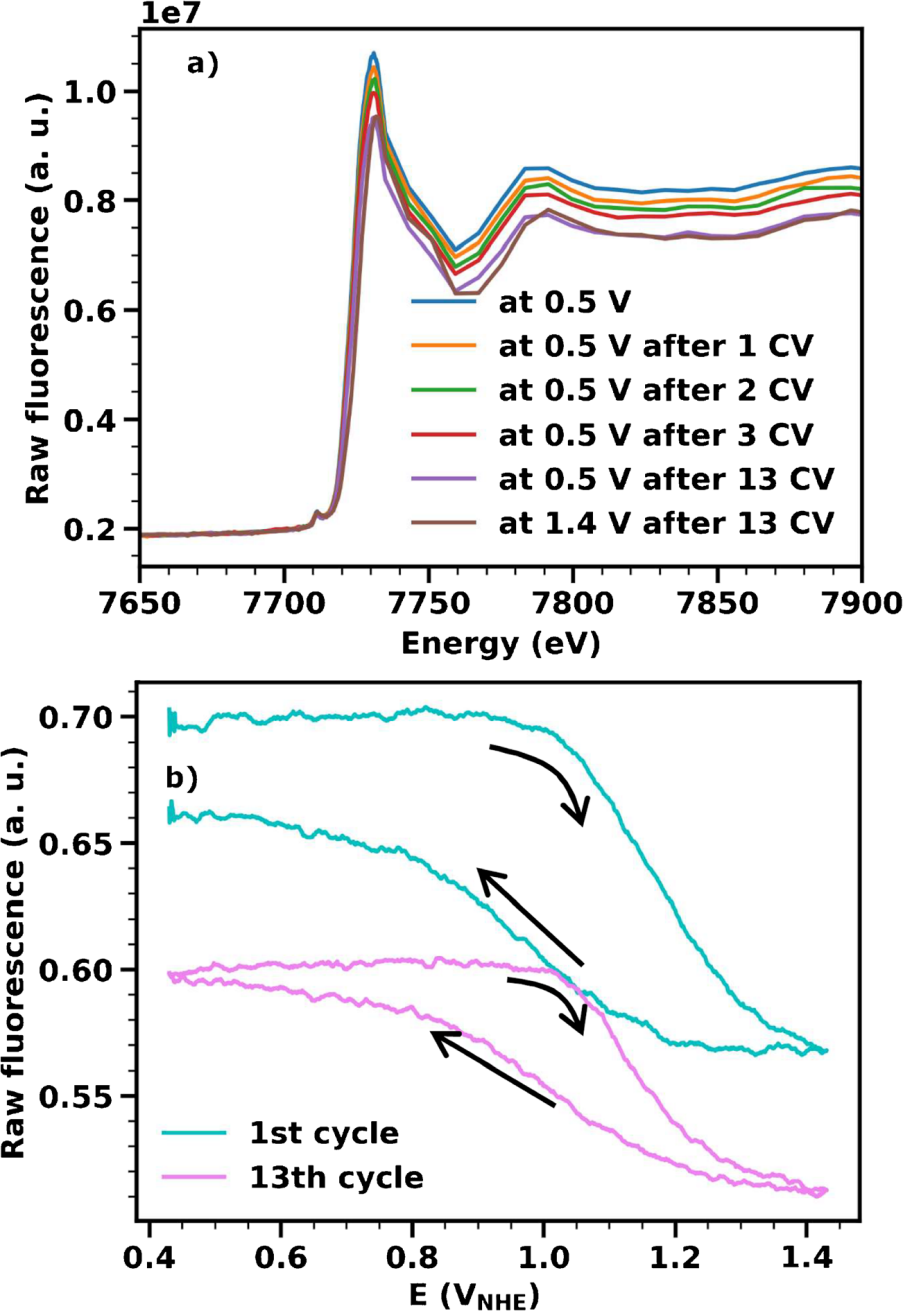
Effect of sample dissolution on the fluorescence signal for a CoCat sample (thickness 180 nm, corresponding to 25 mC cm^−2^) operated in KPi at pH 7. **a** Non-normalized XANES spectra (fluorescence divided by incoming beam signal) recorded at the beginning of the experiment as well as after different numbers of CVs. **b** Fluorescence excited at 7722 eV recorded during the 1st and 13th CVs (detailed explanation of the experiment in the text)

For certain types of samples (including CoCat), dissolution effects cannot be avoided completely. Thus, it is important to correct for dissolution, in order to obtain reliable oxidation state values from the fluorescence. In the following, we will present a simpler and still effective procedure for dissolution corrections, after discussing quantitatively the amount of film dissolved. We recorded four operando XANES spectra at 0.5 V_NHE_, interspersed with single CV measurements; during the CVs, the incoming X-ray beam was fixed at 7722 eV. This sequence was followed by 10 additional CVs and, lastly, two XANES spectra measured at 0.5 V_NHE_ and 1.4 V_NHE_ (Fig. 4). The list of performed XANES measurements is available in Table 1. The decrease in the intensity of the fluorescence signal for each subsequent XANES spectrum, well visible at energies above 7800 eV, reflects the level of sample dissolution during each preceding CV (Fig. 4a). The dissolution effect is also visible in the fluorescence signal recorded during the CVs and causes a mismatch of the start and end fluorescence values (Fig. 4b, blue trace). After 13 CVs, the effect of dissolution is virtually absent (Fig. 4b, magenta trace).

In order to quantify the amount of film dissolution, the fluorescence signal recorded during the XANES measurements was divided by the incoming X-ray intensity and a background was subtracted by fitting a straight line in the region before the edge rise. From this dataset, the signal intensity for energies above 7820 eV was measured and the amount of the dissolved film was calculated, as a percentage of the initial fluorescence intensity (Table 1). During the first CV, 3% of the catalyst is dissolved, but the dissolved amount decrease to 0.5% per CV after repeated cycling. The shift in edge position between subsequent XANES measurements reveals that, in addition to dissolution, a fraction of Co atoms is irreversibly oxidized during the series of CVs, causing a change of about 0.1 oxidation-state units during the sequence of 13 CVs. The minor significance of this irreversible oxidation can be estimated, by comparing it to the amplitude of the total oxidation state change (0.64 units), which is observed in the potential range of a CV. The value of 0.64 oxidation state units was obtained as the difference in oxidation state between the XANES spectra recorded at 0.5 V_NHE_ and at 1.4 V_NHE_ (last two spectra in Table 1).

**Fig. 5:**
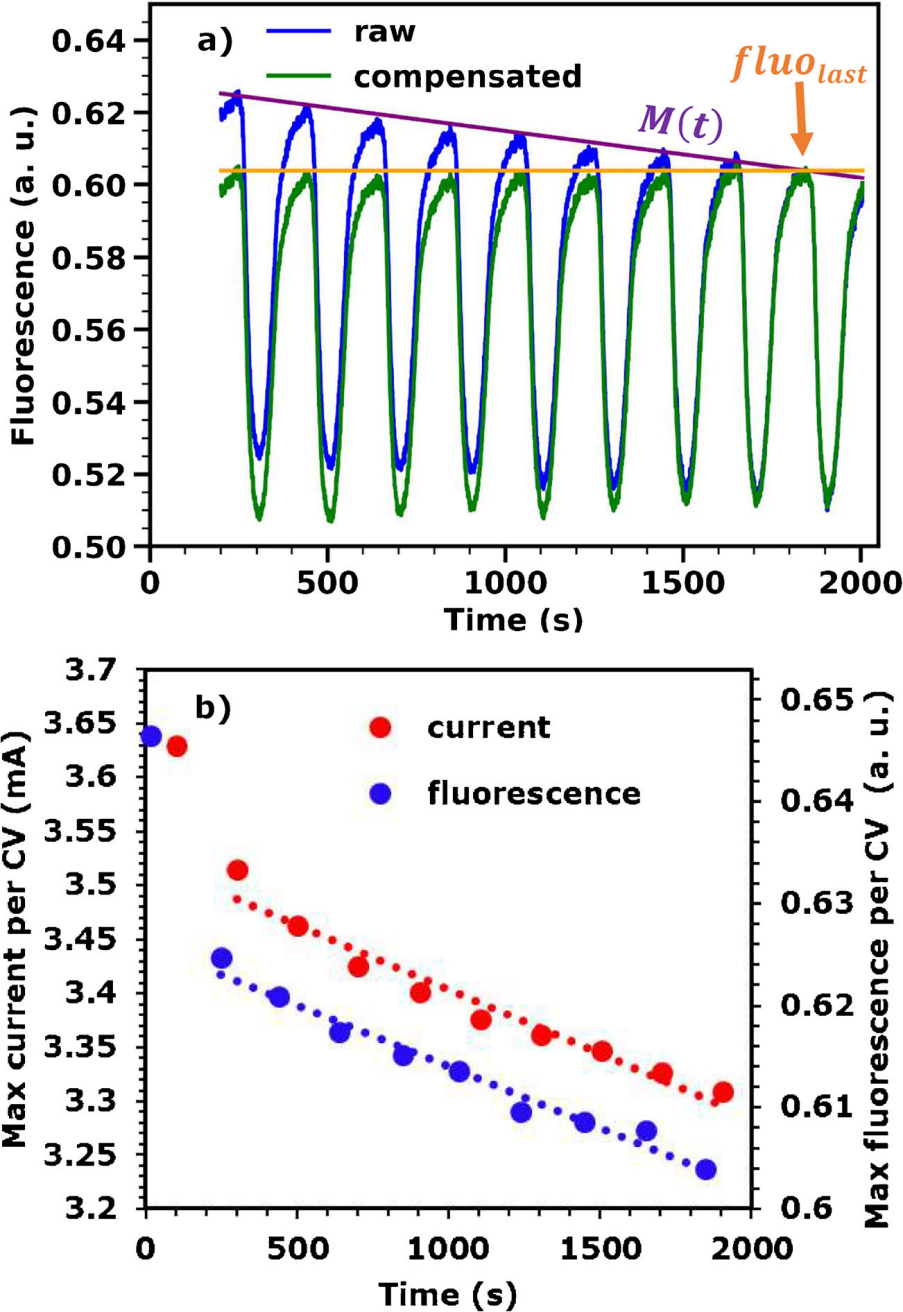
Correction of dissolution during 9 subsequent CVs. **a** A straight line is drawn through the maximum fluorescence value of each CV and used to scale the signal down to the level of the last CV (details in the text). **b** Maximum values reached by the current and the fluorescence signal (raw fluorescence from panel **a**) in each CV. When the first CV is excluded, the decrease in current and fluorescence during the last 9 CVs can be well approximated with a straight line (dotted lines)

In the following, the procedure to correct for the effect of film dissolution is illustrated using the series of 10 subsequent CVs of the described experiment. If we exclude the first CV, the fluorescence and current values decrease linearly with cycle number (Fig. 5b, maximum values reached in each cycle is shown). Thus, it is assumed that, after a certain number of CV cycling, the rate of dissolution is linear over time. Furthermore, we decide to neglect the relatively small effect of the applied potential on the dissolution rate (see Fig. 3 for its relative magnitude) and not to distinguish the irreversible oxidation state change from dissolution, since in our case it is very small, i.e., less than 0.04 units per 10 CVs (see Table 1). In Fig. 5a, the fluorescence signal recorded during 9 CVs is plotted as a function of time (blue trace) and a straight line is drawn through the maximum fluorescence value of each CV (“maximum line,” *M*(*t*)). Since the XANES spectra at 0.5 V_NHE_ and 1.4 V_NHE_ used for mapping between the raw fluorescence signal and formal oxidation state are recorded after the measurement, the dissolution correction consists in scaling down the signal from all CVs to the level of the last CV (Fig. 5a, green trace). To achieve this, the fluorescence signal at each point in time, fluo_meas_(*t*), is divided by the “maximum line” value in that point, *M*(*t*), and then multiplied by the fluorescence value recorded at the end of the CV series (last maximum in the fluorescence, fluo_last_).

1 $$ {\mathrm{fluo}}_{\mathrm{cor}}(t)=\frac{{\mathrm{fluo}}_{\mathrm{meas}}(t)}{M(t)}\times {\mathrm{fluo}}_{\mathrm{last}} $$

We find that, for the CoCat films investigated here, repeated CV cycling before the start of the experiment reduces the impact of film dissolution on the X-ray fluorescence intensity to less than 10% of the changes resulting from potential-dependent oxidation state changes (see Fig. 3b). In this situation, the simple scaling procedure illustrated above is sufficient to correct for dissolution effects and obtain reliable metal oxidation-state values.

However, if the impact of film dissolution were more severe, it would be necessary to correct for dissolution in a more elaborate way, taking into account the potential dependence of the dissolution rate. For severe, potential-dependent dissolution, appropriate corrections could be obtained in a separate experiment on an identically prepared catalyst film where the fluorescence intensity is recorded for energies of the incoming X-rays well above the X-ray edge region (like the experiment shown in Fig. 3). This fluorescence signal provides a good estimate of the amount of (non-dissolved) Co ions in the catalyst film and can be used for potential-dependent dissolution correction.

### A proof of concept: operando investigation of metal oxidation state during cyclic voltammetry

**Fig. 6:**
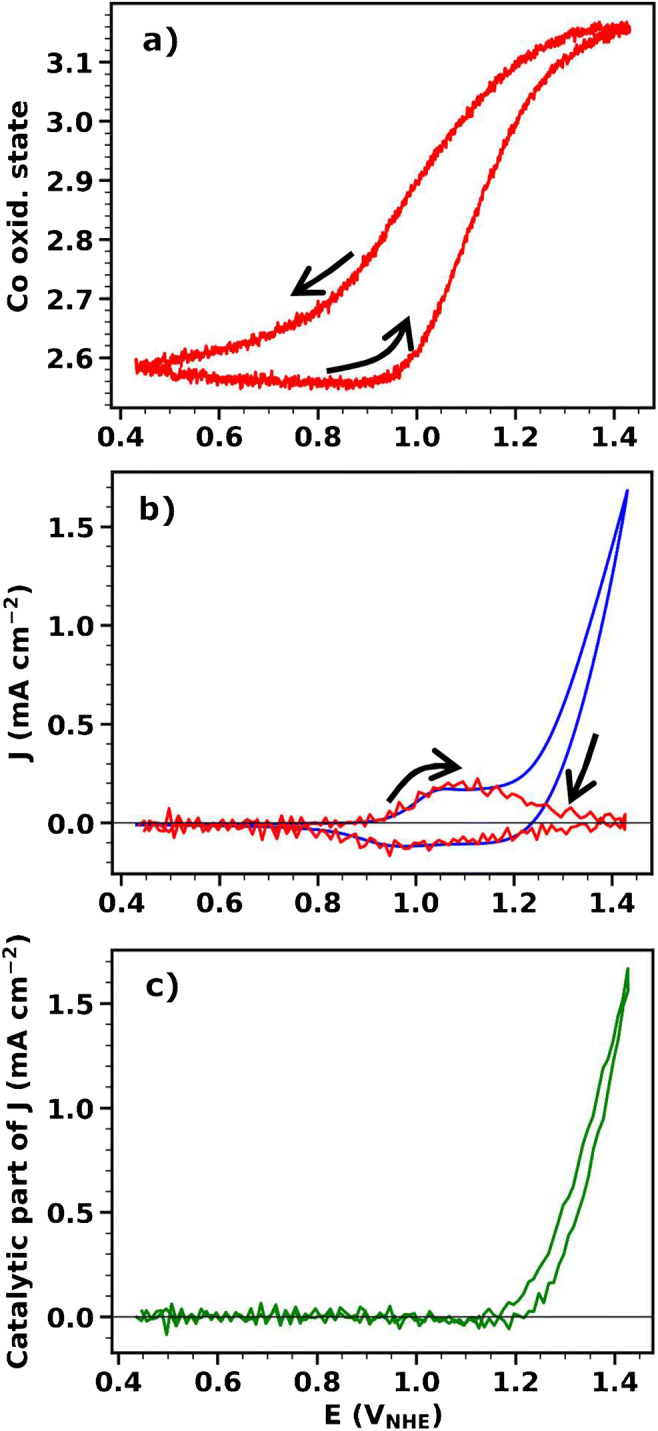
Cyclic voltammetry experiment (scan rate 10 mV s^−1^) for a CoCat sample (thickness 90 nm, corresponding to 12.5 mC cm^−2^) operated in 0.1 M KPi electrolyte at pH 7. Data averaged from 14 CVs are shown. **a** Average oxidation state of the Co ions. For excitation at 7722 eV (center of Co absorption K-edge), the continuously recorded X-ray fluorescence was converted to an average Co oxidation state following the procedure described in Fig. 1. **b** Current density (blue line) and redox current density (i.e., component of the current responsible for oxidation state changes, red line), obtained from the derivative of the fluorescence signal by means of Eq. 2. The dissolution factor (see Eq. 3) was set to 0.7, resulting in excellent agreement between the redox current and the measured electric current in the redox-wave CV region. **c** Catalytic current density calculated as the difference between the total measured current density and the redox current density calculated from the derivative of the oxidation-state signal by means of Eq. 2. The black arrows indicate the scan direction

To illustrate the power of the presented approach, an operando investigation of changes in Co oxidation state was performed during a cyclic voltammetry experiment. CVs for a CoCat film in 0.1 M KPi (pH 7) were carried out with a 10-mV s^−1^ scan rate, while recording the Co K_α_ fluorescence excited in the middle of the Co absorption K-edge (7722 eV) (Fig. 6). After correction for film dissolution, the X-ray fluorescence was converted to absolute Co oxidation states, following the rationale presented in Fig. 1 (Fig. 6a). The changes in the fluorescence signal reflect changes in Co oxidation state and can be mathematically expressed as the time derivative of the fluorescence signal. When the number of Co ions in the investigated metal oxide film is known, the component of the current density which is responsible for Co oxidation state changes (*I*_redox_) can be calculated, according to:

2 $$ {I}_{\mathrm{redox}}=\frac{dQ}{dt}=\frac{d\left(\mathrm{ox}.\mathrm{state}\right)}{dt}\times {Q}_{\mathrm{metal}}=\frac{d\left(\mathrm{ox}.\mathrm{state}\right)}{dV}\times {v}_{\mathrm{scan}}\times {Q}_{\mathrm{metal}} $$

where $\frac{d\left(\mathrm{ox}.\mathrm{state}\right)}{dV}$ is the derivative of the fluorescence signal after conversion to oxidation-state values, *v*_scan_ is the CV scan rate, and *Q*_metal_ is the film deposition charge, which corresponds to the number of Co ions in the Co-based catalyst film times the elementary charge. Since the CoCat film undergoes dissolution, a dissolution factor had to be used in the calculation to obtain sensible results:

3 $$ {Q}_{\mathrm{metal}}={Q}_{\mathrm{deposited}}\times \mathrm{diss} $$

For the data shown in Fig. 6b, diss   =   0.7 was assumed (corresponding to 30% film dissolution). In the present investigations, the value of the dissolution factor was chosen *a posteriori*, for optimal agreement between the measured current and the redox current calculated by Eq. 2. Alternatively, an experimental value for *Q*_metal_ may be obtained by measuring the metal amount in the sample directly after the experiment.

By comparing the derivative of the fluorescence with the current recorded during the CV, we could distinguish between current due to (i) Co oxidation state changes, for which the X-ray fluorescence derivative follows the current, and (ii) catalytic oxygen production, for which the experimental current deviates from the calculated redox current (Fig. 6b). Since no other electron-accepting processes are expected in this potential range, the difference between the current and the derivative of the fluorescence is expected to be the catalytic current, i.e., the current responsible for oxygen production (Fig. 6c).

At non-catalytic potential, a full accordance between the “redox current” (*I*_redox_), obtained from the derivative of the fluorescence signal and the measured current is observed (Fig. 6b). If a second metal is present in the sample, the sum of the derivative of the two fluorescence signals, collected at the respective metals edges, can be compared with the measured current. The accordance between fluorescence derivative and measured current implies that, in this potential range, the only process observed is the oxidation and reduction of the metal. On the contrary, the presence of a discrepancy would have provided an indication for either a charge transfer to other elements in the system (like in the case of catalytic activity) or an “unexpected” characteristic of the redox transition. Examples include a transient major structural change, a spin-state transition or a ligand-centered oxidation/reduction, which can be too fast to be observed by XANES or EXAFS on a minute scale. The comparison of the X-ray signal and the current is a diagnostic tool for such unforeseen transitions.

The collected data for CoCat show that, at low potential, there is a mixture of Co^II^ and Co^III^ ions in the structure of CoCat. The wave in the CV, observed in the anodic scan at ca. 1.05 V_NHE_, corresponds to Co^II^ → Co^III^ oxidation. At 1.15 V_NHE_, there is the onset of catalytic oxygen production, which is paralleled by accumulation of Co^IV^ ions in the material. At higher current densities, changes in the oxidation state are slowed down and tend towards saturation. An interesting observation is that, during the cathodic scan, the oxidation state is higher compared to the anodic scan at the same potential, indicating the presence of a slow component in the Co reduction process. Time-resolved experiments, where fluorescence at 7722 eV is recorded during potential jumps, would facilitate further understanding of these processes.

## Discussion

The dependence of the metal oxidation state on the applied potential provides important information when researching new catalysts for water oxidation; this information can be obtained by operando XAS techniques. A clear limitation of standard XAS techniques is that they require the collection of full XANES spectra, which necessitates specially dedicated equipment or takes several minutes at standard beamlines. Slow XANES data collection also implies that the catalyst is operated under steady-state conditions, where no oxidation-state or structural changes take place during data collection. This severely limits the time resolution and essentially excludes the possibility to study the dynamics of fast oxidation state changes as a response to potential changes, e.g., during CVs or potential jumps, protocols often used in electrochemistry to study the mechanism of the catalytic reaction.

In this study, we presented a method that allows monitoring the oxidation state of metal-containing electrocatalysts during electrochemical operation, by using standard XAS equipment. (We employed a double-crystal monochromator and a scintillation detector installed at a bending magnet beamline XPP-KMC-3 [31] at the BESSY II third-generation synchrotron.) Advantages, requirements, and limitations of this method are summarized in Table 2.

**Table. 2:** Summary of the advantages, requirements, and limitations of the single-energy hard/tender X-ray absorption method presented in this study.

Advantages	Requirements and limitations
Events tracked with (sub)millisecond time resolution	Reasonable sample stability, operation on X-ray transparent electrode
Element specificity combined with bulk sensitivity	No surface-specific sensitivity (typ. beam penetration depth > 100 μm)
Calibrated estimates of oxidation states	Availability of reference compounds
Moderate X-ray beam brilliance suffices, thereby limiting radiation damage	Tunable X-ray excitation energy (typically) provided by a synchrotron radiation source

The method is based on the detection of X-ray fluorescence using a single (fixed) X-ray excitation energy [8, 28, 37, 39, 40]. In this work, an analytical procedure is presented to obtain time-resolved metal oxidation states from the X-ray fluorescence signal using two calibration curves. The first calibration curve provides the relation between raw fluorescence signal and absorption K-edge energy position and the second curve provides the relation between K-edge energy position and metal oxidation state. This approach allows us, using standard equipment for fluorescence-detected XAS, to achieve time resolution down to the millisecond (and potentially also microsecond) time range, with a reliability comparable to technically clearly more challenging methods, like energy-dispersive XAS [20, 21] or QXAFS spectroscopy [19, 23, 24]. Although promising regarding time-resolved experiments, there are also noteworthy limitations of energy-dispersive XAS and QXAFS. The QXAFS is limited by (i) the mechanical stability of the monochromator, which can affect the spectral quality, and (ii) the scanning speed, the latter limiting temporal resolution. Energy-dispersive XAS is applicable only for experiments in transmission mode, which often hampers the performance of operando experiments. The single-energy XAS fluorescence monitored during electrochemical experiments does not have these disadvantages. In addition, it is far more accessible, because it can be easily implemented on every XAFS bending magnet beamline equipped with a standard monochromator.

Monitoring the X-ray fluorescence excited at a fixed energy during a CV was already demonstrated in 2013 and the method was called FEXRAV (fixed energy X-ray absorption voltammetry) [28]. In the present study, we make a step further and illustrate that the X-ray fluorescence signal can be used to quantify the absolute oxidation state during a CV. We describe a procedure to determine the absolute oxidation state and present a systematic investigation of possible problems and strategies to avoid data misinterpretation. The described approach can also be extended to other electrochemical techniques like chronoamperometry (potential jumps) and chronopotentiometric measurements.

Possible sources of errors are X-ray-induced radiation damage or natural structural instability of the sample (e.g., loss of contact to the supporting conducting electrode) under studied electrochemical conditions. These effects need to be eliminated in advance to perform reliable operando XAS measurements. Other sources of systematic errors can be compensated by adequate data analysis. These include possible issues with proper energy calibration, overlapping reversible and irreversible structural changes in the catalyst during operando measurements, and sample dissolution in the electrolyte. The complex procedure used to convert the X-ray signal into an oxidation state values is also a source of error, the error added by the different steps of the procedure is discussed in ESM Note 4 and Fig. S8. For the proof-of-concept experiment presented in this manuscript, we estimated an error of ± 0.05 oxidation state units, when comparing two experiments performed with the same equipment and the same set of reference compounds (relative error), while the uncertainty range estimated for the absolute oxidation state value is ± 0.09 oxidation state units.

Here, we present as an example a study of Co oxidation state changes during CVs in CoCat operated at neutral pH. These experiments allowed to detect metal-centered redox transitions during cyclic voltammetry and calculate the current responsible for these transitions, identify the catalytic component of the current, assess whether all redox transitions are metal-centered, and measure the average oxidation state at which water oxidation begins. In particular, comparing the derivative of the X-ray fluorescence (measured at a single excitation energy) with the measured current allows to discern more complex behaviors of a redox transition, such as transient major structural change, spin-states transition, or ligand-centered oxidation/reduction. This has been previously observed for NiFe oxides using UV/vis absorption, where a discrepancy between the measured current and the derivative of the absorption signal was tentatively assigned to ligand oxidation [41]. In comparison to UV/vis absorption, the proposed X-ray technique is less sensitive to external factors that hardly can be addressed quantitatively (e.g., light scattering effects) and offers quantitative estimate of metal ion oxidation states as well as comparison between electric current densities and X-ray signals.

The single-excitation-energy XAS method has already been used in various electrochemical experiments [28], e.g., jumps between oxidizing and reducing potentials to study redox kinetics [8, 37] and hole accumulation [10], or cyclic voltammetry in mixed metal oxides [40, 42]. It opens up new perspectives for a variety of future, highly informative experiments to investigate the mechanisms of electrocatalytic water oxidation and other electrocatalytic processes.

## Supplementary information

ESM 1 (PDF 1.33 mb)
