# Supplementary material for: Operando tracking of oxidation-state changes by coupling electrochemistry with time-resolved X-ray absorption spectroscopy demonstrated for water oxidation by a cobalt-based catalyst film
Source: Anal Bioanal Chem. 2021 Jul 17;413(21):5395–408. doi: 10.1007/s00216-021-03515-0 (PMC8405515; doi:10.1007/s00216-021-03515-0)
Supplement: Supplementary file 1 — (PDF 1.33 mb) [file 216_2021_3515_MOESM1_ESM.pdf]

Supplementary Information - Journal of Analytical and Bioanalytical Chemistry

Operando tracking oxidation-state changes by coupling of electrochemistry with time-resolved X-ray absorption spectroscopy demonstrated for water oxidation by a cobalt-based catalyst film

Chiara Pasquini [1], Si Liu [1], Petko Chernev [1,2], Diego Gonzalez-Flores [1,3], Mohammad Reza Mohammadi [1,4], Paul Kubella [1], Shan Jiang [1], Stefan Loos [1,5], Katharina Klingan [1], Vadim Sikolenko [1,6], Stefan Mebs [1], Michael Haumann [1], Paul Beyer [1], Luca D'Amario [1,2], Rodney D. L. Smith [1,7], Ivelina Zaharieva [1,] * and Holger Dau [1,] *

[1] Department of Physics, Freie Universität Berlin, Arnimallee 14, 14195 Berlin (Germany)
[2] present address: Department of Chemistry - Ångström Laboratory, Molecular Biomimetics, Uppsala University, Lägerhyddsvägen 1, 75120 Uppsala, Sweden
[3] present address: Centro de Electroquímica y Energía Química (CELEQ) and Escuela de Química, Universidad de Costa Rica, 11501 2060, San José, Costa Rica
[4] present address: Department of Physics, University of Sistan and Baluchestan, Zahedan, 98167-45845 (Iran)
[5] present address: Fraunhofer Institute for Manufacturing Technology and Advanced Materials (IFAM), Winterbergstraße 28, 01277, Dresden (Germany)
[6] present address: Karlsruhe Institute of Technology, Karlsruhe (KIT), Adenauerring 20, 76131 Karlsruhe (Germany)
[7] present address: Department of Chemistry, University of Waterloo, 200 University Ave. W, N2L 3G1 Waterloo, ON (Canada)

E-mail addresses: ivelina.zaharieva@fu-berlin.de; holger.dau@fu-berlin.de

The file includes:

Supplementary Note 1. Determining the edge position: the integral method

Supplementary Note 2. Energy calibration

Supplementary Note 3. X-ray radiation damage

Supplementary Note 4. Accuracy of the conversion procedure

Fig. S1 Scheme of the operando setup

Fig. S2 Full set of XANES spectra recorded at different potentials (addition to Fig. 1a, b)

Fig. S3 XANES spectra of Co containing reference compounds

Fig. S4 and S5 Energy calibration of XANES spectra

Fig. S6 Time-resolved experiment to estimate X-ray radiation damage

Fig. S7 Example of structural modification observed during the experiment

Fig. S8 Experimental reproducibility and estimation of the accuracy

Supplementary Note 1. Determining the edge position: the integral method

Various methods have been used to describe the X-ray edge position by a single energy value, the edge energy. Edge energies are usually determined as the half-height of the normalized X-ray edge-rise (energy where the fluorescence intensity of the normalized edge is 0.5), or as the energy of the main inflection point of the X-ray edge spectrum (energy of the steepest fluorescence rise) [1, 2]. Both methods have the advantage that are intuitively clear and mathematically straightforward to implement. However, the determined edge energy position is often affected by edge shape changes related to structural changes of the material and are not necessarily coupled to oxidation state changes. The alternative option used here is the 'integral method' that derives the X-ray edge position, via suitable integration procedure of the entire edge rise, as detailed elsewhere [3]. The advantage of the 'integral method' is that the resulting edge energy value: (i) is largely insensitive to edge-shape changes (as they may arise due to changes in the coordination environment), (ii) reflects the mixing ratio well for mixed-valent compounds, and (iii) is less sensitive to noise contributions (because the integration effectively involves averaging over a large number of data points) such that it can be determined reliably, without potentially critical data smoothing protocols, also for X-ray data with comparably high noise contributions [3].

Supplementary Note 2. Energy calibration

The energy of the X-rays at most synchrotron beamlines is calculated from the position of the monochromator, often with high relative precision, but typically with significant uncertainties regarding the absolute energy scale. The absolute energy scale may depend on experimental factors like positioning of mirrors and monochromator elements, slit widths and heat load to elements of the beamline optics. Thus, in typical X-ray absorption experiments, an energy calibration procedure is performed, ideally based on the simultaneous measurement of the sample of interest and of a stable reference compound. The reference compound must have a known position of the absorption edge and it is often a metal foil placed behind the sample. (For metal foils, edge position have been determined on an absolute energy scale and are available as tabulated values [4]). However, this procedure introduces some constraints in the experimental set-up. In the case of a large electrochemical cell which is not transparent for X-rays, like the one used in this work, the measurement of a reference compound placed behind the sample is technically impossible (see Figure S1).

Therefore, an alternative procedure for energy calibration was developed, which is based on the alignment of the pre-edge rise. The pre-edge is a feature of the XANES spectrum of cobalt ions, whose position, unlike the position of the main absorption edge, is not strongly influenced by the oxidation state of the sample [5]. The spectra of reference compounds, used to build the calibration curve, are also aligned following the same rationale. Since pre-edges are aligned, what we are practically using for calibration is the distance between pre-edge rise and edge rise.

The alignment here was facilitated by simulating the derivative of the pre-edge feature with Gaussian functions (see Supplementary Information, Figure S4), the center of the Gaussian function, corresponding to the middle of the pre-edge rise was set to 7708 eV. Since the pre-edge is not perfectly well described by a Gaussian function, the range of the fit slightly affects the final alignment. The variation in oxidation state due to different fit-ranges used for energy calibration is illustrated in the Supplementary Information (Figure S5), showing an offset in the oxidation states of 0.06 and an overall conserved trend. This value represents the sensitivity of the absolute oxidation-state estimates towards an uncertainty in the calibration of the energy axis.

Other approaches are feasible, for example, the fine-tuning of the energy axis, aiming at optimal alignment of the pre-edge rise of the measured spectrum and a reference spectrum. For X-ray data of sufficiently high quality, optimal alignment can be judged by mere visual inspection at high precision. We emphasize that, whatever method is used, the pre-edge alignment requires extreme care, because its precision is a major determinant of the precision of the final oxidation-state estimate.

Supplementary Note 3. X-ray radiation damage

A possible source of misleading results is radiation damage in form of X-ray photoreduction of high-valent metal sites, which is often observed when exposing a sample to strong X-rays for prolonged time periods. This problem has been extensively discussed elsewhere [6]. In this study, to avoid possible radiation damage to the sample, an out-of-focus geometry (with 11 x 2 mm beam spot size) and a low X-ray beam intensity (about 10^{11} photons/s [7]) were used. Furthermore, an additional control experiment was performed, to rule out the presence of radiation damage: the incoming X-ray beam energy was fixed in the middle of the absorption edge (E_{exc} = 7722 eV) and the X-ray fluorescence was monitored over the course of several

minutes (Figure S6). During this time, the X-ray beam was switched off and on again. The fluorescence signal slightly decreases over time, which can be due either to photoreduction or sample dissolution. However, the rate of the decrease is independent on whether the X-ray beam is off or on; no indications for sharp changes in the film behavior exists, suggesting that photoreduction is not involved. The same experiment was repeated with the energy set well above the absorption edge (E_{exc} = 8400 eV) and a similar result was obtained. At this excitation energy, the X-ray fluorescence is not sensitive to the metal oxidation state but only to the amount of fluorescing material in the film, suggesting that the decrease in fluorescence is assignable to sample dissolution. Accordingly, the presence of extensive radiation damage, meaning photoreduction of the metal centers caused by the X-ray beam, can be excluded in our case.

Supplementary Note 4. Accuracy of the conversion procedure

The analytical procedure proposed for the conversion of the X-ray fluorescence signal into an oxidation state requires a rather complex data analysis. In this paragraph, we analyze the potential inaccuracy that is added by the different steps of the procedure and estimate a confidence interval for the results obtained in the proof-of-concept.

The procedure is composed by two conversion step: (1) the conversion of the fluorescence signal into an edge energy-position, based on the reference XANES measurements performed during each experiment (first calibration line) and (2) the conversion of the edge energy-position into an oxidation state, based on reference compounds (second calibration line). The absolute value of the oxidation state (absolute accuracy) is affected by both conversions and therefore has a lower accuracy, while the comparison between different experiments, using the same set of reference compounds (relative accuracy), is more reliable.

An estimation of the relative accuracy is provided by a repetition of the experiment (Fig. S8a). The experiment was repeated using the same methodology and instrumentation, during a separate measuring sessions: with new beam alignment and set-up. The sample used during the second repetition was twice as thick as the one used in the first repetition and provides an estimation of the impact of sample thickness on the results. The thicker sample started from an oxidation state of 2.66 and reached 3.09, while the thinner sample started from an oxidation state of 2.58 and reached 3.16. Likely part of this difference is due to certain Co atoms in the thicker sample not changing their oxidation state. From this result we can estimate a 0.05

oxidation state confidence interval when comparing two experiments analyzed with the same method, same sample thickness and same set of reference compounds.

Three factors contribute to the absolute accuracy: (1) the relative accuracy, (2) the error on the "second calibration line", used for conversion of edge positions into oxidation state values, and (3) the error made in the alignment of the energy axis, between the experimental XAS spectra and the reference compounds XAS spectra.

To obtain the "second calibration line" the edge position of the reference compounds in function of their oxidation state is simulated with a linear equation ($y = m x + q$), Fig. 1d, and the standard deviation (σ) on each of the fit parameter is obtained. Two alternative calibration lines were calculated using the standard deviation: $y = (m + \sigma_m) x + q – \sigma_q$ and $y = (m - \sigma_m) x + q + \sigma_q$. This two alternative calibration lines were employed for the conversion procedure on an exemplary experiment, obtaining different final values for the Co oxidation state during the experiment (Fig. S8b). The variation in the Co oxidation state caused by the use of an alternative "second calibration line" is 0.03 oxidation state unit, we use this value as an estimate on the error caused by the "second calibration line". It is important to note that the reference compounds used for this study were carefully chosen and fit well on a line, using a different set of reference compounds can lead to higher inaccuracies.

The error made in the alignment of the energy axis is discussed in the Supplementary Note 2. A slightly alternative approach is used for alignment resulting in a different oxidation state value, the variation amounts to 0.06 oxidation state units. This value is assumed to be the error caused by the alignment of the energy axis.

By summing all the different contributions, we obtain an estimate for the confidence interval on the oxidation state absolute value (absolute accuracy) of 0.15 oxidation state units.

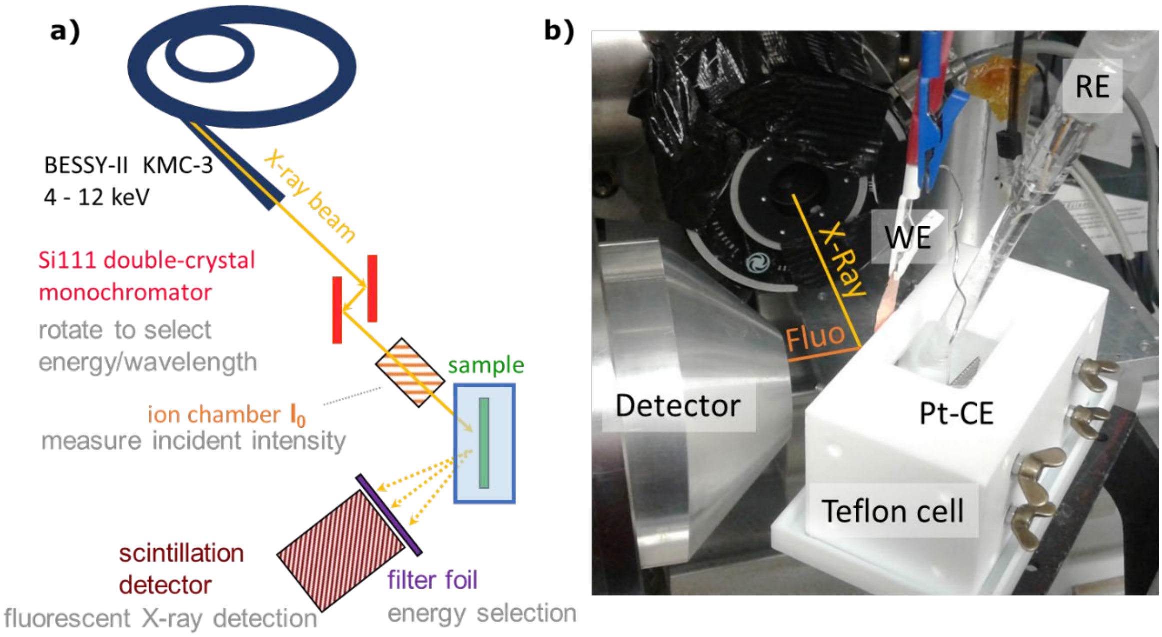

Fig. S1 Operando setup used at KMC-3-Beamline at BESSY II synchrotron radiation facility, Helmholtz Zentrum Berlin. a) Scheme of the beamline setup and geometry used for registration of X-ray fluorescence. b) Picture of the homemade electrochemical cell used for operando XAS experiments.

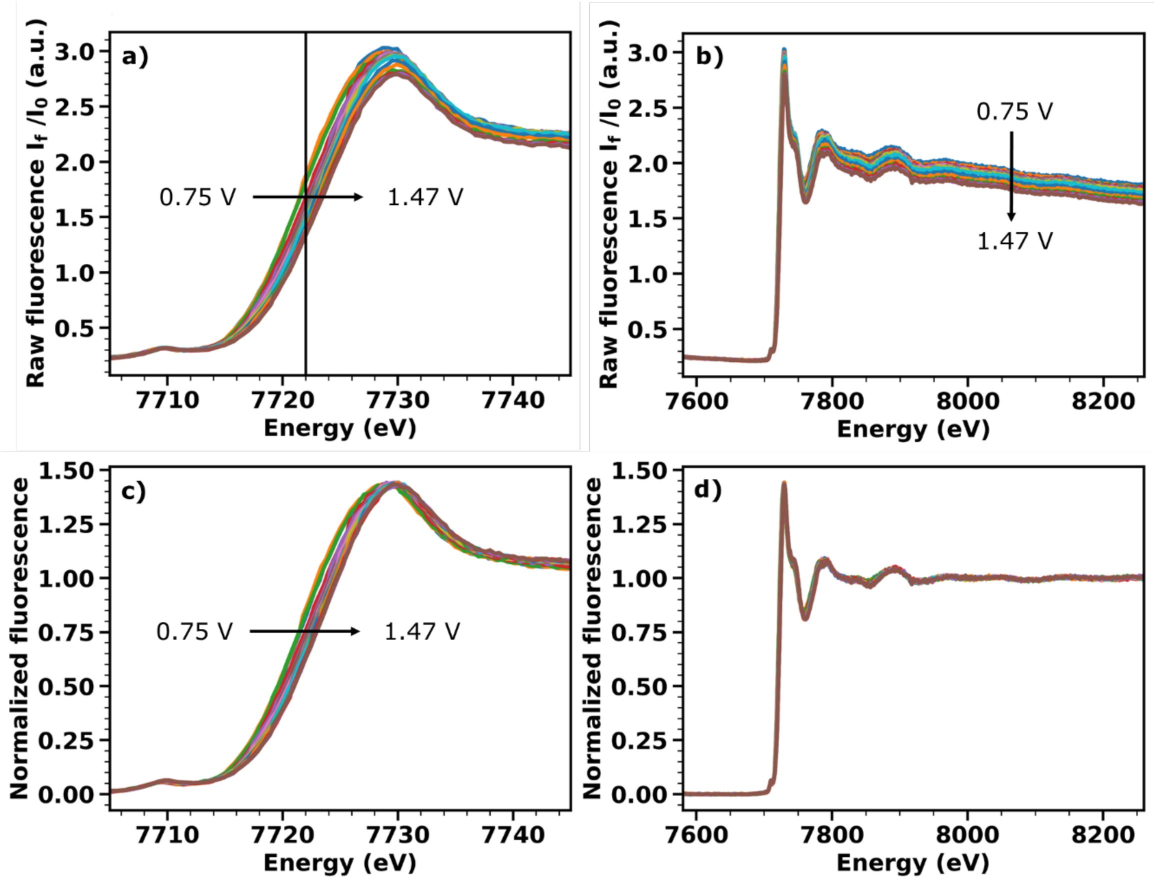

Fig. S2 Calibration Co K-edges recorded operando at different applied potentials (0.75 to 1.47 V_{NHE}) for thin CoCat films (20 mC cm^{-2}) operated in 0.1 M KPi solution at pH 7. (a-b) Raw fluorescence: the recorded X-ray fluorescence signal was divided by the incoming beam intensity, then the value at 7722 eV was extracted. (c-d) The edges were further processed by subtracting a background, obtained by fitting a straight line in the energy region between 7587 and 7685 eV, and normalizing the intensity after the edge to 1, via division by a straight line fitted in the energy region between 7735 and 8282 eV. Edge position was determined from the normalized fluorescence using the integral method [3]. The value at 7722 eV determined from panel (a) vs. the edge position determined from panel (c) are reported in Fig. 1c of the main text.

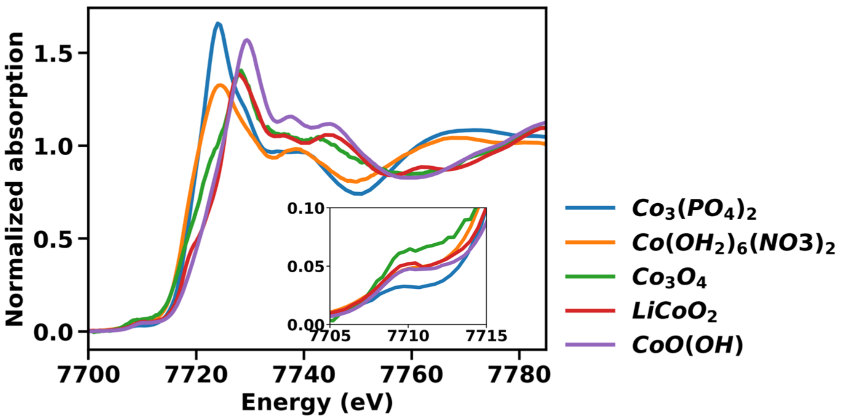

Fig. S3 Normalized Co K-edges for reference compounds of known oxidation state. In the inset, a magnification of the pre-edge region is shown. The edge position extracted using the integral method [3] vs. Co oxidation state is reported in Fig. 1d in the main text.

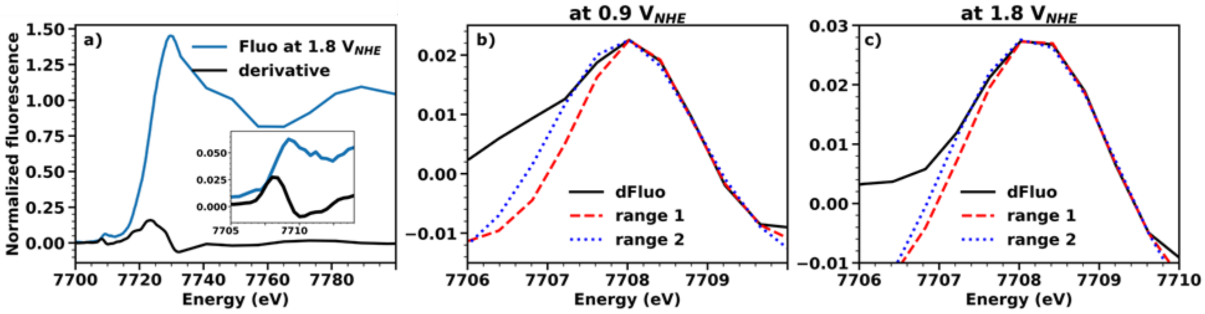

Fig. S4 Details on the XANES alignment procedure. The derivative of the edge was calculated (a), then the derivative peak corresponding to the rise of the pre-edge was simulated with the sum of a Gaussian function and a linear slope (b-c). XANES spectrum was shifted on the energy axis such that the center of the Gaussian (pre-edge rise) is at 7708 eV. Since the shape of the low-energy side of the derivative of the pre-edge is varying a lot between different spectra, sometimes being considerably wider, two fit approaches were considered. Range 1: the fit is centered symmetrically around the maximum, considering only the high-energy part. Range 2: the entire peak was simulated. The effect of the fit on the estimated Co oxidation state is shown in Fig. 3 in the main text. Range 1 was used consistently for the alignment of spectra presented in the other figures.

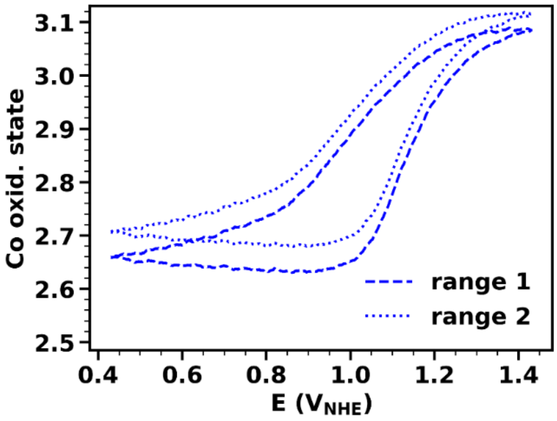

Fig. S5 Impact of the edge alignment step on the accuracy of the fluorescence conversion to oxidation state. For a CV scan (scan rate 10 mV/s) on a CoCat film (25 mC cm^{-2}) operated in 0.1 M KPi buffer at pH 7. The two curves represent the result of using two different ranges for the Gaussian fit of the derivative of the pre-edge feature (simulations shown in Figure S5). The use of a different fit range causes a variation of 0.06 oxidation state units. Data averaged from 9 CVs are shown after smoothing with a moving average over 15 points. The sample used here is thicker than the one used in Fig. 6a, a comparison between the two is presented in Fig. S8.

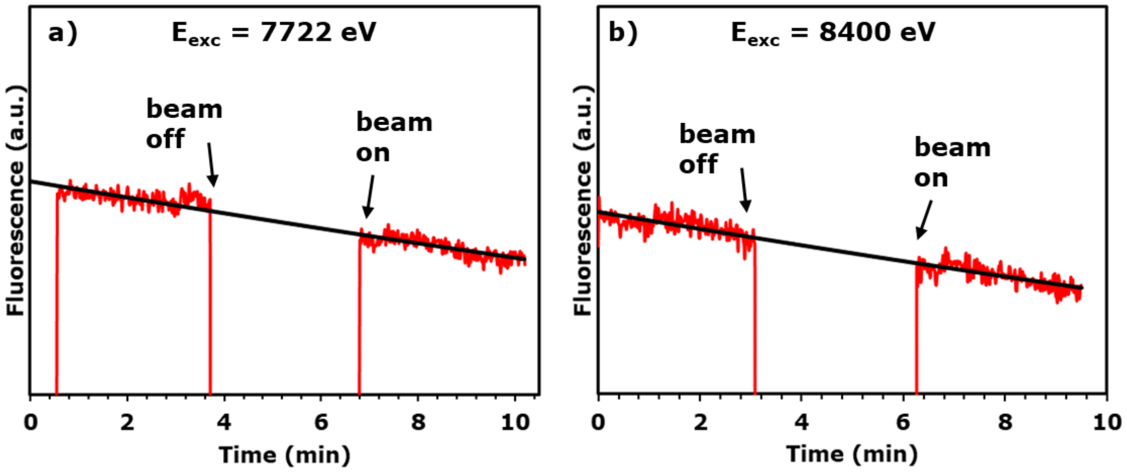

Fig. S6 Time course of the fluorescence excited at (a) E_{exc} = 7722 eV (middle of the absorption edge) or (b) E_{exc} = 8400 eV (above the edge), for a CoCat film kept at open circuit potential in 0.1 M KPi at pH 7. During the measurement the beam was shut down for approx. 3 min. The fluorescence was simulated with an exponential decay function according to the equations: $y = 59\ e^{-0.014x} + 293$ for panel (a) and $y = 41\ e^{-0.016x} + 238$ for panel (b).

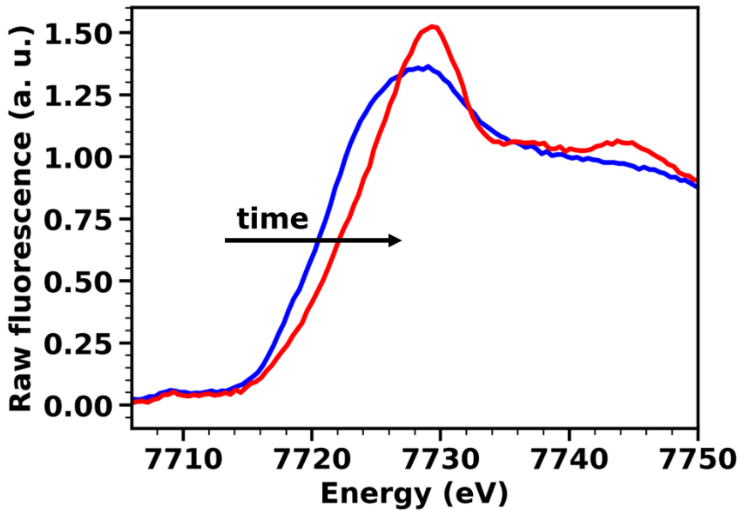

Fig. S7 Example of modification of the edge shape over the course of an operando experiment. XANES were recorded at the Co K-edge for a dry CoCat film (blue line) and for the same film after operation in 1 M KOH (red line). The change in the edge shape is due to a modification of the sample structure.

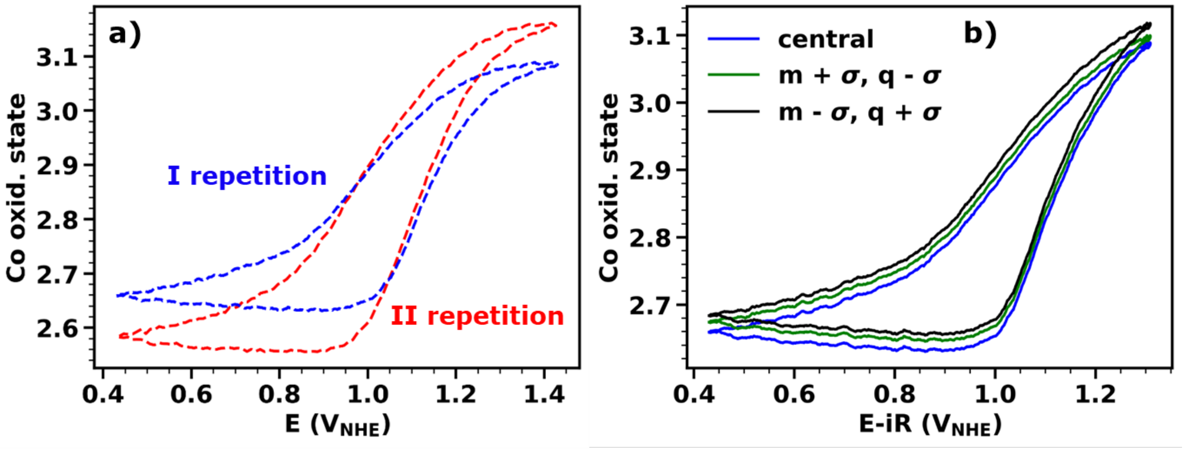

Fig. S8 Reproducibility and estimation of absolute accuracy for fluorescence conversion to oxidation state during a CV scan (scan rate 10 mV/s) for a CoCat film operated in 0.1 M KPi buffer at pH 7. Data averaged from 9 CVs are shown. (a) The reproducibility of the experiment and the impact of film thickness on results is investigated. The same CV experiment is performed during two different measuring sessions (i.e. beam-times) and for two different film thicknesses (blue line 25 mC cm^{-2} and red line 12.5 mC cm^{-2}). The differences between the two repetitions amount to ca. 0.08 oxidation state units on both the lower and upper values. (b) Impact of the choice of reference compounds on the absolute accuracy of the calculated oxidation state. The second calibration line (Fig. 1d) is the one based on the reference compounds and used to link the edge position to the metal oxidation state. The m and q parameters (y = m x + q) obtained by simulation of this line were flexed using one standard deviation (σ), obtaining two new calibration lines: $y = (m + \sigma_m) x + q - \sigma_q$ and $y = (m - \sigma_m) x + q + \sigma_q$. The graph shows the variation in Co oxidation state during a CV when these new calibration lines are employed for conversion of the edge position to a Co oxidation state. The variation observed is ca. 0.03 oxidation state units.

References

1. Bokhoven JAV, Lamberti C. X-Ray Absorption and X-Ray Emission Spectroscopy. John Wiley & Sons, Ltd; 2016.

2. Hummer AA, Rompel A. Chapter Eight - X-ray absorption spectroscopy: A tool to investigate the local structure of metal-based anticancer compounds in vivo. In: Christov CZ, editor. Advances in protein chemistry and structural biology. Academic Press; 2013. p. 257-305.

3. Dau H, Liebisch P, Haumann M. X-ray absorption spectroscopy to analyze nuclear geometry and electronic structure of biological metal centers—potential and questions examined with special focus on the tetra-nuclear manganese complex of oxygenic photosynthesis. Anal Bioanal Chem. 2003;376(5):562-83. doi:10.1007/s00216-003-1982-2.

4. Thompson A, Attwood D, Gullikson E, Howells M, Kim K-J, Kirz J et al. X-ray data booklet. Berkeley: Lawrence Berkeley National Laboratory, University of California; 2001.

5. Koningsberger DC, Prins R. X-ray absorption: principles, applications, techniques of EXAFS, SEXAFS, and XANES. John Wiley and Sons,New York, NY; 1988.

6. King HJ, Fournier M, Bonke SA, Seeman E, Chatti M, Jumabekov AN et al. Photon-induced, timescale, and electrode effects critical for the in situ X-ray spectroscopic analysis of electrocatalysts: The water oxidation case. J Phys Chem C. 2019;123(47):28533-49. doi:10.1021/acs.jpcc.9b06944.

7. Zizak I, Gaal P. The KMC-3 XPP beamline at BESSY II. Journal of large-scale research facilities. 2017;3:A123. doi:10.17815/jlsrf-3-112.
